# Attitudes of Health Professionals Toward Digital Health Data Security in Northwest Ethiopia: Cross-Sectional Study

**DOI:** 10.2196/57764

**Published:** 2024-11-06

**Authors:** Ayenew Sisay Gebeyew, Zegeye Regasa Wordofa, Ayana Alebachew Muluneh, Adamu Ambachew Shibabaw, Agmasie Damtew Walle, Sefefe Birhanu Tizie, Muluken Belachew Mengistie, Mitiku Kassaw Takillo, Bayou Tilahun Assaye, Adualem Fentahun Senishaw, Gizaw Hailye, Aynadis Worku Shimie, Fikadu Wake Butta

**Affiliations:** 1 Department of Health Informatics, College of Medicine and Health Science, Debre Markos University, Debre Markos, Ethiopia; 2 Department of Health Informatics, School of Public Health, College of Medicine and Health Science, Wollo University, Dessie, Ethiopia; 3 Department of Health Informatics, School of Public Health, College of Medicine and Health Science, Mettu University, Mettu, Ethiopia

**Keywords:** health, profession, digital, attitude, security, data

## Abstract

**Background:**

Digital health is a new health field initiative. Health professionals require security in digital places because cybercriminals target health care professionals. Therefore, millions of medical records have been breached for money. Regarding digital security, there is a gap in studies in limited-resource countries. Therefore, surveying health professionals’ attitudes toward digital health data security has a significant purpose for interventions.

**Objective:**

This study aimed to assess the attitudes of health professionals toward digital health data security and their associated factors in a resource-limited country.

**Methods:**

A cross-sectional study was conducted to measure health professionals’ attitudes toward digital health data security. The sample size was calculated using a single population. A pretest was conducted to measure consistency. Binary logistic regression was used to identify associated factors. For multivariable logistic analysis, a *P* value ≤.20 was selected using Stata software (version 16; StataCorp LP).

**Results:**

Of the total sample, 95% (402/423) of health professionals participated in the study. Of all participants, 63.2% (254/402) were male, and the mean age of the respondents was 34.5 (SD 5.87) years. The proportion of health professionals who had a favorable attitude toward digital health data security at specialized teaching hospitals was 60.9% (95% CI 56.0%‐65.6%). Educational status (adjusted odds ratio [AOR] 3.292, 95% CI 1.16‐9.34), basic computer skills (AOR 1.807, 95% CI 1.11‐2.938), knowledge (AOR 3.238, 95% CI 2.0‐5.218), and perceived usefulness (AOR 1.965, 95% CI 1.063‐3.632) were factors associated with attitudes toward digital health data security.

**Conclusions:**

This study aimed to assess health professionals’ attitudes toward digital health data security. Interventions on educational status, basic computer skills, knowledge, and perceived usefulness are important for improving health professionals’ attitudes. Improving the attitudes of health professionals related to digital data security is necessary for digitalization in the health care arena.

## Introduction

Health care digitalization has empowered and enabled health care professionals to achieve positive health care outcomes [1,2]. The World Health Organization (WHO) has released guidance for digital health after a critical review of available evidence for the benefits, harms, acceptability, feasibility, resource use, and equity considerations of digital health interventions [3]. The growth of digital technology in the health sector has changed the relationship between health care professionals and patients. It helps to bring new attitudes toward health care or medical data. However, these new ways of thinking have been raising concerns about digital security [4].

Digital security in health care has an ultimate impact on an individual’s quality of life [5,6] because the storage, processing, transmission, and analysis of data are major health care activities. In electronic health transactions, the privacy regulations required by the Health Insurance Portability and Accountability Act tried to assure consumers that as their health records became fully electronic and networked, their information would be protected [5]. Therefore, digital health security is vital in the internet age for professionals and organizations to keep their information assets safe [7].

However, there is a matter of using digital health security countermeasures, particularly as sensitive electronically protected health information has been at risk because it is accessed by every health care service in various ways digitally [6]. As mentioned by many studies, among the major impediments to the adoption of digital health by users are security concerns [3,4]. Numerous health care data security breaches have occurred owing to a lack of attitude by the user [8]. Most prominently, digital health data were the victim of phishing, ransomware, and malware [9].

As studies elucidated, the attitudes of health professionals toward digital health data security were prominent factors in determining the consequences of adopting digital technology to secure health care data [3]. According to studies, these attitudes influence people’s comprehension of and use of data-security measures [10,11]. Henceforth, digital health data users desire to know more about security measures [12]. However, human error is the main driving force behind security problems. According to a study by a security organization, about 88% of data breaches are caused by employee error [13]. This revealed that health professionals have an inadequate attitude toward digital data security [14]. Various studies elaborated that the contributing factors to attitudes toward security were age, frequent use of digital technology [15], education, and the types of work manners or jobs [16,17]. Therefore, improving the attitudes of health professionals toward data security is the backbone of health care institutions. Another study suggested that improving their knowledge, computer skills, and education level was the best approach to developing a favorable attitude toward digital security [18-20].

Digital technologies used by health care organizations uniquely have the ultimate purpose of processing lifelong and sensitive data. In resource-limited countries, including Ethiopia, health information technology is at a developmental level; some digital health applications have run in health care settings to ease the handling of health information. These applications are smart care, district health information systems, and wearable and wireless health care devices operating in each health care field. As a result, in resource-limited countries, some health organizations have used digital technology. However, there are limitations to using digital health data security tools regarding health professionals’ attitudes. This issue has not yet been addressed. Therefore, the main aim of this research was to assess health care professionals’ attitudes toward digital data security in a limited-resource country. Identifying the factors associated with attitudes toward digital health data security will serve as a baseline for further studies and guide security strategies for health care digitalization.

## Methods

### Study Design and Settings

This study was conducted at a specialized teaching hospital in a resource-limited country in Northwest Ethiopia. All hospitals play an academic and referral role for more than 3.5 million people. For this study, a cross-sectional study design was an appropriate method to assess health professionals’ attitudes toward digital data security. The study was conducted from May 30 to June 20, 2022, at a specialized teaching hospital in Northwest Ethiopia. Appropriate informed consent was taken to administer the study.

### Study Sample and Eligibility Criteria

The source population of the study was health professionals working in specialized teaching hospitals. Health professionals who had used digital devices in those hospitals were selected for the sample population. On the contrary, health professionals who had no experience in digital health and who were not in health care settings during the period of data collection were excluded from the study.

### Sample Size Determination and Sampling Procedure

**Figure 1.**
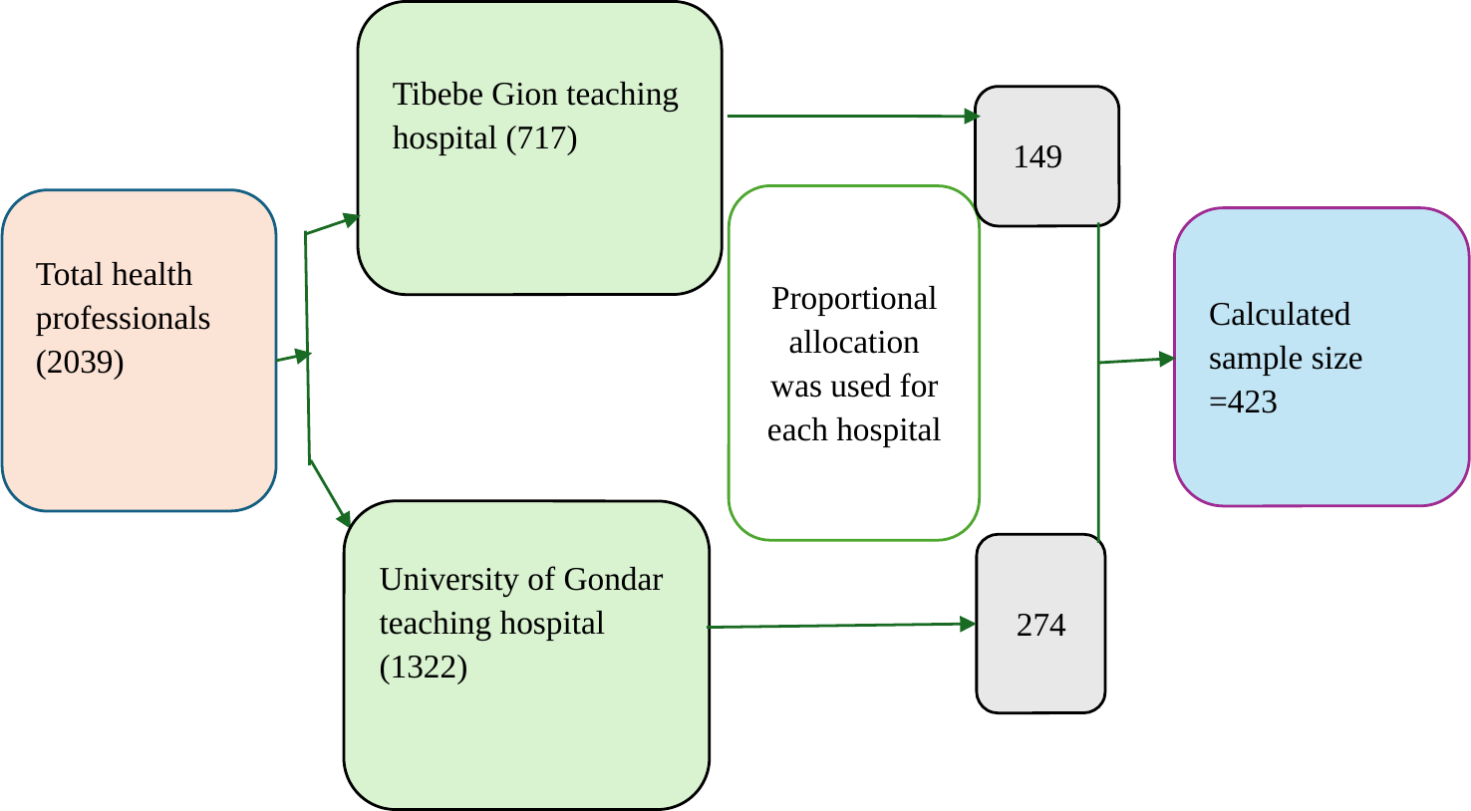
Sampling procedure of the study

The sample size was determined using single-population proportions and a nonresponse rate of 10%. The sample size was calculated assuming a 95% significance level, 5% marginal errors, and a 50% population proportion, which is recommended by biostatistician experts [21]. In this case, the total sample size was 423. Proportional allocations were made for each health care institution. Then, based on the sampling frame of the list of health professionals obtained from human resources management, simple random sampling was used to pick the study participants who were working in specialized teaching and referral hospitals. These steps are illustrated in Figure 1.

### Study Variables

The outcome variable of this study was the attitudes of health professionals toward digital health data security. Other characteristics such as sociodemographics (age, gender, educational status, work experience, and monthly salary), behavioral and technological characteristics (computer skills, knowledge of digital health data security, use of the internet, use of social media, perceived ease of use, and perceived usefulness), and organizational characteristics (training and health care workload) were the explanatory variables of the study.

### Measuring Instruments

Digital health data security refers to digital health data techniques to safeguard health care data from illegitimate or unauthorized access, alteration, and destruction.

Knowledge of digital health data security refers to the know-how of health professionals to use security tools while processing health data, which we measured using a 10-item Likert scale. The data were not normally distributed, as shown using the Shapiro-Wilk test (*P* value <.05), skewness, and kurtosis. Therefore, knowledge was determined based on the median; a score above the median value of 35 (IQR 31‐40) indicated good knowledge, and a score below the median score indicated poor knowledge [22,23].

Attitudes toward digital health data security refer to the feelings of health professionals about digital security tools (data encryption, antivirus, etc) and while processing health data using digital devices, which have been measured using a 10-item Likert scale. The data were asymmetrically distributed and categorized by the median. A score above the median value of 31 (IQR 27‐33) indicated a favorable attitude, and a score below the median indicated an unfavorable attitude [15,23,24].

### Data Collection Tool and Quality Assurance

A structured self-administered questionnaire was adopted and modified using information from various publications [15,22,24-27]. The questionnaire was written in the English language. A pretest was conducted to ensure reliability before conducting the study. Trained medical professionals collected the data. Finally, the investigators took over overall supervision. Validated forms were then completed using statistical software to minimize errors and verify missing values or incompleteness. To ensure the quality of our surveys, we conducted research consultations and a pretest to check the tool. Based on suggestions from medical experts, word corrections were made to facilitate comprehension, and Cronbach α scores of 60% or higher were used. The pretest showed that the Cronbach α scores of attitude and knowledge were 0.83 and 0.86, respectively.

### Statistical Analysis

Data were input into EpiData version 4.6 software (EpiData Association) and exported to Stata (version 16; StataCorp LP). *χ*^2^ assumptions, collinearity, and other assumptions, such as incomplete values, were checked and tested by presenting the data via summary statistics. After generating the cleaned data, further analysis was performed using the Stata software. The means, medians, SDs, percentages, and frequencies were used to illustrate the results in tables and graphs. We also used binary logistic regression to identify the relevant factors. Variables with *P* values less than or equal to .20 were candidates for multivariate analysis. Multicollinearity was checked using the variable inflation rate. Finally, an odds ratio with a 95% CI and a *P* value less than .05 were used to determine the outcome variable by adjusting for confounding effects. Model fit was checked using the Hosmer-Lemeshow test.

### Ethical Considerations

Ethical approval was received from the Institutional Review Board of the University of Gondar, College of Medicine and Health Science, the Institution of Public Health Ethical Review Committee (no. IPH/2013/2014). The procedures used in this study obey the rules of the Declaration of Helsinki. A formal letter was received from each specialized teaching hospital unit coordinator (no. 1061/90/13). Finally, written consent forms were delivered with each questionnaire, and appropriate written consent was taken from the respondents.

## Results

### Sociodemographic Characteristics

Of the 423 health professionals, 95% (n=402) participated in this study, whereas 5% (n=21) of participants did not complete the survey due to various situations. In terms of sociodemographic characteristics, out of 402 health professionals, 254 (63.2%) were male, and 148 (36.8%) were female, with a mean age of 34.5 (SD 5.87) years. Among all study participants, 131 (32.6%) were aged between 20 and 30 years, 214 (53.2%) were in the range of ages between 31 and 40 years, and 57 (14.2%) were above 40 years. In the case of marital status, 166 (41.3%) were single, 191 (47.5%) health professionals were married, and the remaining 45 (11.2%) were others who might be separated or widowed. Regarding educational status, 293 (72.9%) health professionals had bachelor’s degrees, 55 (13.7%) had master’s degrees, and 54 (13.4%) had postmaster’s degrees (specialist and subspecialist). Further, 224 (55.7%) and 178 (44.3%) health professionals had 1-5 years of work experience and above 5 years of work experience, respectively. Regarding monthly salary, the majority of health professionals were paid below 10,000 Ethiopian birr (US $82.95; Table 1).

**Table 1.** Sociodemographic characteristics of health professionals’ attitudes on digital health data security at specialized teaching hospitals in Northwest Ethiopia in 2022 (n=402).

Variables	Frequency	Percent
**Gender**		
Male	254	63.2
Female	148	36.8
**Age (years)**		
20‐30	131	32.6
31‐40	214	53.2
>40	57	14.2
**Marital status**		
Single	166	41.3
Married	191	47.5
Other^a^	45	11.2
**Educational status**		
Bachelor degree	293	72.9
Master’s degree	55	13.7
Postmaster’s degree^b^	54	13.4
**Work experience**		
1‐5 years	224	55.7
>5 years	178	44.3
**Monthly salary in Ethiopian birr (1 Ethiopian birr=US $0.008)**		
≤10,000	273	67.9
>10,000	129	32.1

a Other (separated, widowed).

b Postmaster’s degree (specialist, subspecialist).

### Behavioral and Organizational Characteristics

Based on organizational characteristics, the results illustrate that 58% (n=233) of health professionals agreed that there is a workload in the health care organization, while 42% (n=169) agreed that there is no workload in the health care settings. In the case of digital training, 57% (n=229) of health professionals had taken basic computer training, whereas 43% (n=173) of the participants had not taken training delivered by their health care institutions. In terms of behavioral characteristics, 54.7% (n=220) of health professionals perceived digital health security tools as easy to use, while 45.3% (n=182) perceived digital health security tools as not easy to use. Regarding usefulness, 23.1% (n=93) of health professionals perceived digital health security tools as useful to use, whereas 76.9% (n=309) perceived that their usefulness was not adequate for digital health data security, as shown in Table 2.

**Table 2.** Behavioral, organizational, and technological characteristics of health professionals regarding digital health data security at specialized teaching hospitals in Northwest Ethiopia in 2022 (n=402).

Variables	Frequency	Percent
**Health care workload**		
Low workload	169	42
Have workload	233	58
**Use internet**		
Yes	394	98
No	8	2
**Use social media**		
Yes	390	97
No	12	3
**Taken computer training**		
Yes	229	57
No	173	43
**Trained in digital data security**		
Yes	75	18.7
No	327	81.3
**Digital literacy**		
Poor literacy	148	36.8
Good literacy	254	63.2
**Perceived ease of use**		
Not easy	220	54.7
Easy	182	45.3
**Perceived usefulness**		
Inadequate usefulness	309	76.9
Usefulness	93	23.1

### Technological Characteristics

The study shows that according to technical characteristics, 63.2% (n=254) of health professionals were digitally literate, whereas 36.8% (n=148) of the participants did not have adequate digital literacy. Concerning digital security, 18.7% (n=75) of health professionals were trained in digital security, whereas the majority of participants, which accounted for 81.3% (n=327), were not adequate in digital security. Related to internet and social media accessibility, the majority of health professionals, which accounted for 98% (n=394), had used the internet, and 97% (n=390) had used social media. A few participants had limited access to the internet and social media (Table 2).

### Health Professionals’ Attitudes Toward Digital Health Data Security

#### Overview

The first and main aim of this study was to find out the prevalence of health professionals’ attitudes toward digital health data security in a limited-resource country. Using the median score, those above the median value of 31 (IQR 27‐33) have favorable attitudes, whereas those below the median value have unfavorable attitudes [23]. Based on that, from a total of 402 health professionals, 245 participants had a favorable attitude, which accounted for 60.9%. In contrast, 157 (39.1%) participants had an unfavorable attitude toward digital health data security countermeasures. This indicates that out of 5 health professionals, 3 need an intervention to improve their attitudes and reach an optimal level of security to minimize risk for digital health data.

#### Factors Associated With Attitudes Toward Digital Health Data Security

According to the context of a limited-resource country, the significant factors of health professionals’ attitudes toward digital health data security were identified using logistic regression analysis. After identifying the variable using binary logistic analysis, the candidate variables were adjusted for final analysis and interpretations. Therefore, education status, computer skills, knowledge, and perceived usefulness were determined as associated factors for health professionals’ attitudes toward digital health data security.

This study revealed that educational status was a significant factor and that the professionals with a postmaster’s degree were 3.29 (adjusted odds ratio [AOR] 3.292, 95% CI 1.16‐9.34) times more likely to have a favorable attitude toward digital data security, and this difference was statistically significant at a *P* value of .03. Second, health professionals with basic computer skills were 1.8 (AOR 1.807, 95% CI 1.11‐2.938) times more likely to have a favorable attitude toward digital data security, with a statistically significant *P* value of .02. The findings also show that health professionals with good knowledge of data security were 3.238 (AOR 3.238, 95% CI 2.0‐5.218) times more likely to have a favorable attitude toward digital data security, with a statistically significant *P* value of 0.00. Similarly, the professionals who perceived the usefulness of digital data security were 1.965 (AOR 1.965, 95% CI 1.063‐3.632) times more likely to have a favorable attitude toward digital health data security, with a statistically significant at a *P* value of .03 (Table 3).

**Table 3.** Bivariable and multivariable analysis of factors associated with the attitudes toward digital health data security among health professionals working at specialized teaching hospitals in Northwest Ethiopia (n=402).

Variables	Unfavorable attitude, n (%)	Favorable attitude, n (%)	COR^a^ (95% CI)	AOR^b^ (95% CI)
**Educational status**				
BSc degree	130 (32.3)	163 (40.6)	1	1
Master’s	21 (5.2)	34 (8.5)	1.29 (0.715‐2.33)	0.96 (0.488‐1.886)
Postmaster’s	6 (1.5)	48 (11.9)	6.38 (2.65‐15.37)	3.292 (1.16‐9.34)^c^
**Monthly salary in Ethiopian birr (1 Ethiopian birr=US $0.008)**				
≤10,000	121 (30.1)	152 (37.8)	1	1
>10,000	36 (9)	93 (23.1)	0.763 (0.563-1.035)	1.166 (0.646‐2.105)
**Taken computer training**				
No	85 (21.1)	88 (21.9)	1	1
Yes	72 (17.9)	157 (39.1)	2.106 (1.4‐3.168)	1.498 (0.936‐2.397)
**Use email**				
No	15 (3.7)	13 (3.2)	1	1
Yes	142 (35.3)	232 (57.7)	1.885 (0.872‐4.078)	1.01 (0.426-2.4)
**Basic computer skills**				
Poor	85 (21.1)	63 (15.7)	1	1
Good	72 (17.9)	182 (45.3)	3.41 (2.23‐5.217)	1.807(1.11-2.938)^c^
**Knowledge of digital data security**				
Poor	115 (28.6)	91 (22.6)	1	1
Good	42 (10.5)	154 (38.3)	4.634 (2.99‐7.182)	3.238 (2.009-5.218)^d^
**Perceived usefulness**				
Inadequate usefulness	136 (33.8)	173 (43)	1	1
Useful	21 (5.2)	72 (17.9)	2.695 (1.578‐4.604)	1.965 (1.063-3.632)^c^
**Perceived ease of use**				
Not easy	103 (25.6)	117 (29.1)	1	1
Easy	54 (13.4)	128 (31.8)	2.087 (1.38‐3.156)	1.289 (0.789-2.106)

a COR: crude odds ratio.

b AOR: adjusted odds ratio.

c *P*<.05.

d *P*<.01.

## Discussion

### Principal Findings

The main aim of this study was to assess health professionals’ attitudes toward digital health security in health care in a limited-resource country. The proportion of health professionals who had a favorable attitude toward digital health data security was 60.95% (95% CI 56.0%‐65.6%). While comparing this finding with resourceful countries however, this was contrary to studies conducted in Portugal [4], Norway [14], Turkey [28], and Sweden [29]. The majority, which accounted for 81.7% (1283/1569) of the participants, had a favorable attitude, which entails that health professionals have trust in data security techniques and believe that worthy health needs virtuous digital data security measures. In rich countries, there may be sufficient resources to give training on digital health security techniques and be actively involved in health care technology compared with resource-limited country.

The findings of this study identified educational status as a significant factor; professionals with postmaster degrees were 3.29 times more likely to have a favorable attitude toward digital data security. This denotes that the level of health professional educational status has an enormous effect on attitude toward digital data security techniques. This finding is in line with the studies conducted in European countries that show education status has a significant effect on attitudes toward digital health data security [16]. This is because, through the life of education, nearly developed digital security tools are accessed by health data users to be authenticated.

Professionals with basic computer or digital literacy were 1.807 times more likely to have a favorable attitude toward digital data security. This suggests that taking basic literacy courses related to digital technology influences a favorable attitude toward the use of digital security techniques. This finding is evidenced in Turkey, where acquiring digital literacy skills has improved attitudes toward data security [30]. However, in this study, only 63.2% (254/402) of health professionals had good computer skills. This indicates that there was a low level of involvement of health professionals in the use of digital technology to facilitate daily health care tasks.

The findings also showed that the knowledge of health professionals is 3.238 times more likely to have a favorable attitude toward digital data security. This revealed that health professionals’ knowledge of digital security significantly influences their attitude toward digital data security, and this is consistent with a study in Norway that found that the knowledge of health care professionals significantly enhances their attitude in health care settings [14]. Similarly, a study conducted in Indonesia indicates that the workforce’s knowledge of digital security has a beneficial effect on their attitude, resulting in a strong and enthusiastic data security culture [20].

Finally, professionals who perceived the usefulness of digital data security were 1.965 times more likely to have a favorable attitude toward digital data security. This explains why well-perceived usefulness in digital security has a significant association with attitudes toward digital health data security. This result is supported by a study conducted in Korea that used the Technology Acceptance Model, which showed that perceived usefulness made a great difference in digital security [31].

Overall, the contribution of this study to the research on attitudes toward digital health security is as follows. First, the study revealed that the education level and digital literacy of health professionals have positively influenced their attitudes toward digital health data security. Second, perceived usefulness and knowledge about data security are also important to improve their attitude toward digital data security. These findings are essential and contribute to studies on digital health.

### Strength and Limitation

Digital health security is an urgent issue in the digital health environment. Especially, the findings of health professionals’ attitudes toward digital data security are the basis for expanding the installation of digital health in resource-limited countries. Therefore, the findings of this study will serve as a guide for interventions in digital health development. This cross-sectional study may have introduced bias in identifying the cause-and-effect relationship.

### Conclusions

According to the results of this study, the attitudes of health professionals toward digital health data security in a limited-resource country were inadequate. To improve the attitudes of health professionals, working on the significant factors, which are updating educational status, improving computer skills and knowledge toward digital security, and increasing perceived digital health security usefulness, is the basis for improving attitudes and ultimately has the purpose of safeguarding digital health data. Therefore, improving health care professionals’ attitudes toward digital data security will guide the development of health care digitalization.

The findings will be forwarded to referral hospitals, the Regional Health Bureau, the Federal Minister of Health, and other institutions. The outcomes may help to improve health professionals’ attitudes toward digital health data security by motivating them to improve their computer skills and enhance their educational level. Health care institutions shall be changing the attitudes of health professionals toward digital data security to create a safe digital environment. Finally, the Regional Health Bureau and the Minister of Health will formulate a plan to ensure the optimum use of digital security measures.

## Abbreviations

AOR: adjusted odds ratio
WHO: World Health Organization
